# Prognostic and diagnostic significance of lncRNAs expression in cervical cancer: a systematic review and meta-analysis

**DOI:** 10.18632/oncotarget.18323

**Published:** 2017-05-31

**Authors:** Shuqi Chi, Lina Shen, Teng Hua, Shuangge Liu, Guobing Zhuang, Xiaoxiao Wang, Xing Zhou, Guozhen Wang, Hongbo Wang

**Affiliations:** ^1^ Department of Obstetrics and Gynecology, Union Hospital, Tongji Medical College, Huazhong University of Science and Technology, Wuhan 430022, China

**Keywords:** cervical cancer, long noncoding RNA, prognosis, diagnosis, meta-analysis

## Abstract

Long noncoding RNAs (lncRNAs) have been reported to be abnormally expressed in cervical cancer (CC) and presumably serve as diagnostic or prognostic markers. We thus performed a systematic review and meta-analysis to evaluate the clinical values of dysregulated lncRNAs in CC. A literature search was performed using the electronic databases PubMed, Embase, and Web of Science. A total of 22 relevant studies were eligible, including 21 on clinicopathological features, 18 on prognosis, and 4 on diagnosis. For clinicopathological features, HOTAIR expression was positively associated with tumor size (odds ratio [OR]=2.19, 95% confidence interval [CI] 1.42-3.38, *P*=0.000) and lymph node metastasis (OR=6.04, 95% CI 3.51-10.42, *P*=0.000). For the prognostic values, up-regulated HOTAIR had an unfavorable impact on overall survival ([OS]; hazard ratio [HR]=1.94, 95%CI 1.17-3.22, *P*=0.011) and disease-free survival (HR=2.61, 95%CI 1.35-5.05, *P*=0.004), and high PVT1 expression was correlated with shorter OS (HR=1.66, 95%CI 1.21-2.29, *P*=0.002). For the diagnostic values, the pooled result showed an area under the curve (AUC) of 0.85, with 85% sensitivity and 81% specificity in discriminating patients with CC from healthy controls. Overall, we conclude that lncRNAs might serve as promising indicators for prognostic and diagnostic evaluation of patients with CC.

## INTRODUCTION

Cervical cancer (CC) is the fourth most commonly diagnosed cancer and the fourth leading cause of cancer-related death among females worldwide, with 527,600 new female cancer cases and 265,700 deaths worldwide in 2012 [1]. With the improvement of diagnostic techniques and therapeutic strategies, the incidence and mortality rates of CC has decreased [2]. However, the overall prognosis of CC patients still remains poor, especially in developing countries [3]. Currently, there are few factors that can be applied to effectively predict the incidence and mortality rates of cancer patients because of the numerous and complex risk factors for CC. Squamous cell carcinoma antigen (SCC-Ag) [4] is a commonly used marker for diagnosis, but low sensitivity and specificity limit its utility. Under such circumstances, it can be clinically challenging to determine novel biomarkers for prognosis and diagnosis of CC.

During the past decades, there has been an explosive growth in knowledge regarding lncRNAs in the field of RNA biology. LncRNAs are broadly defined as RNA molecules greater than 200 nt in length, lacking an open reading frame [5], and they are regulators of gene expression at the chromatin-organizational, transcriptional and post-transcriptional levels [6]. Accumulating evidence has demonstrated that lncRNAs play a non-negligible role during the process of proliferation, migration, and invasion of tumor cells [7-10]. Recently, a relationship between the expression of particular lncRNAs and the survival of cancer patients has also been increasingly reported, especially HOTAIR, a highly oncogenic lncRNA in numerous human malignancies. Furthermore, a large number of observational studies have been carried out to investigate the prognostic and diagnostic role of certain lncRNA in CC lately. To date, a large number of observational studies have been performed to investigate the prognostic and diagnostic role of certain lncRNAs in CC. For example, Yang et al. [11] found that serum expression of the lncRNA PVT1 is higher in CC patients, with 71.6% sensitivity and 98.8% specificity. Another study reported that the lncRNA XLOC_010588 was significantly down-regulated in CC patients and was associated with poor prognosis [12]. With the aim of synthesizing the results of these studies to gain better insight into the clinical value of lncRNAs, we performed a systematic review and meta-analysis to quantify the predictive efficacy of lncRNAs in the aspects of clinicopathological features, prognosis and diagnosis in CC patients.

## RESULTS

### Study selection and characteristics

**Figure 1:**
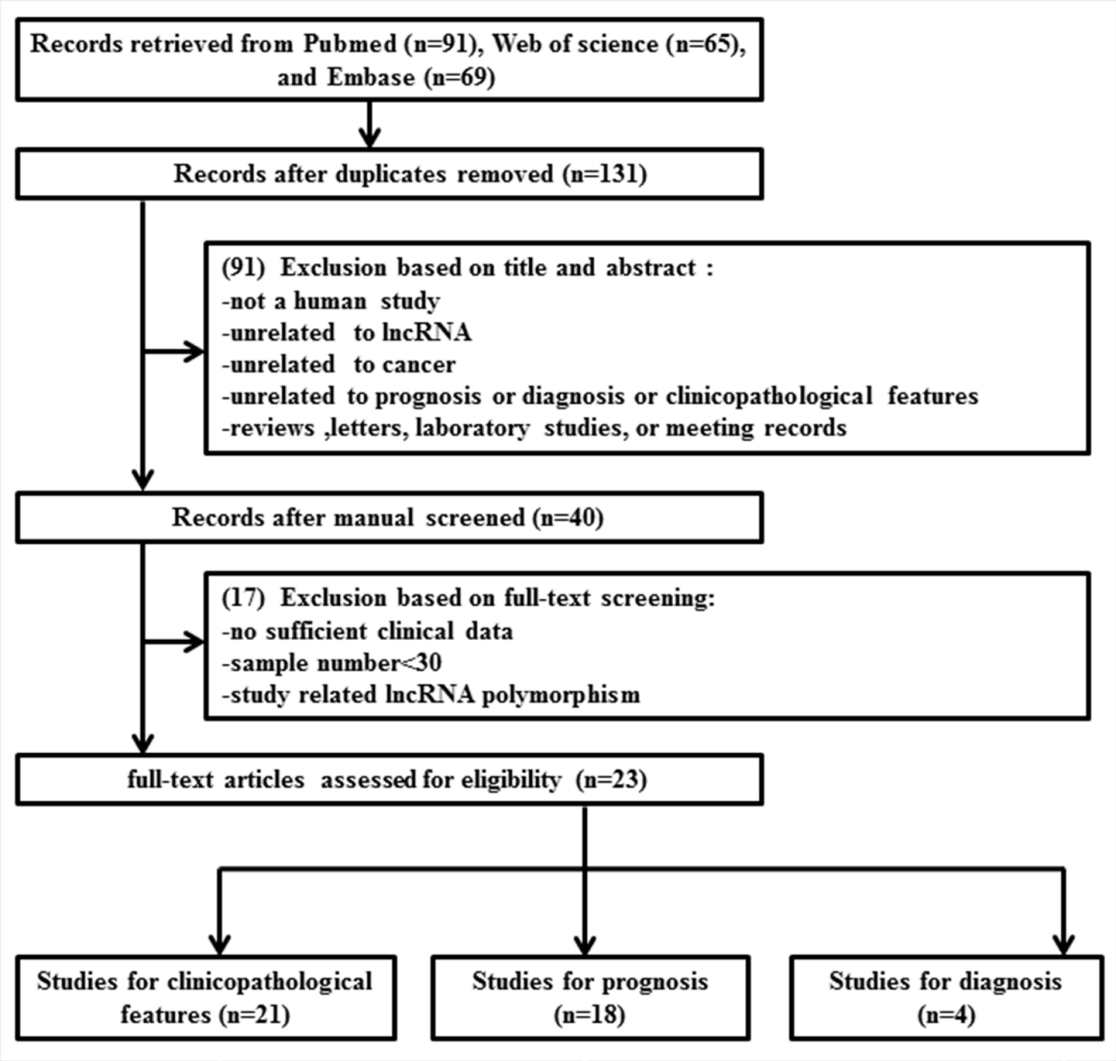
Flow chart of the literature search and selection

As shown in the flow diagram (Figure 1), 225 records were initially identified from PubMed, Embase and Web of science. After screening the titles and abstracts of these studies, 185 duplicate or irrelevant articles were excluded. Subsequently, the remaining 40 full-text articles were assessed for eligibility, and 17 studies, including 14 without sufficient clinical data, 1 with less than 30 samples, and 2 with discussions on lncRNA polymorphism, were further excluded on the basis of the exclusion criteria. No additional studies were identified through our manual search of references from published studies, relevant reviews, and previous meta-analyses. As a result, 22 eligible studies [11-32] encompassing 2363 patients were included in this systematic review and meta-analysis.

All the selected studies were published between 2014 and 2017, including 21 on clinicopathological features, 18 on prognosis and 4 on diagnosis. Most of the studies were from China (77.3%), followed by Korea (13.7%), Japan (4.5%) and America (4.5%). Quantitative real-time polymerase chain reaction (qRT-PCR) assays were used to quantify the lncRNAs in all of the studies. Specimens were composed of tissue (*n* = 20) and serum (*n* = 2). Of the total 15 lncRNAs, 4 (HOTAIR, MALAT1, PVT1 and MEG3) were investigated by at least two studies, and the remaining 11 lncRNAs were studied in a single report. Additionally, 94.44% of the NOS scores for the included studies on prognosis were ≥7 (Supplementary Table 1), and all of the Quality Assessment of Diagnostic Accuracy Studies-2 (QUADAS-2) scores for studies on diagnosis were ≥4, indicating a high quality for most of the studies (Supplementary Table 2).

### Clinicopathological features

Twelve lncRNAs from 21 studies were available to evaluate the effect of their expression on clinicopathological features. The expression of HOTAIR [13-17], CCAT2 [18], CCHE1 [19], MALAT1 [20, 21], SPRY4-IT1 [22], HOXA11-AS [23], HULC [24], PVT1 [11, 25], ANRIL [26] and TUG1 [27] were up-regulated, while the expression of GAS5 [28], XLOC_010588 [12], LET [29], MEG3 [30] and XIST [31] were down-regulated in CC patients. Only a small number of studies reported that dysregulated lncRNAs were related to the level of SCC-Ag and lymphovascular space invasion; most of the studies reported that lncRNAs were significantly associated with tumor size, International Federation of Gynecology and Obstetrics (FIGO) stage and lymph node metastasis (Table 1).

**Table 1:** Summary of the comparison for the p values of the association between lncRNAs and clinicopathological features

studies	LncRNAs	Population	Case number	Cut-off	Expression	Age	Tumor size	Histology	FIGO stage	Differentiation	Lymph node metastasis	Scc-Ag (µg/l)	Lymphovascular space invasion
Cao 2014	GAS5	Chinese	102	median	down-regulation	0.187	0.386	0.851	NA	0.462	NA	NA	NA
Huang 2014	HOTAIR	Chinese	218	median	up-regulation	0.02	0.006	0.686	<0.0001	0.519	<0.0001	0.724	NA
Liao 2014	XLOC_010588	Chinese	218	median	down-regulation	0.336	<0.0001	0.686	<0.0001	0.273	0.07	0.006	NA
Chen 2015	CCAT2	Chinese	123	median	up-regulation	0.415	0.514	NA	0.003	NA	NA	NA	NA
Jiang 2015	LET	Chinese	94	mean	down-regulation	0.867	0.929	0.732	NA	0.057	0.004	NA	NA
Kim 2015	HOTAIR	Korean	111	fold-change	up-regulation	0.8809	0.8839	0.2334	0.7671	NA	0.0437	NA	0.6351
Yang 2015	CCHE1	Chinese	182	median	up-regulation	0.374	<0.001	0.466	0.002	0.432	0.283	0.004	NA
Yang 2015	MALAT1	Chinese	104	median	up-regulation	0.43	0.005	0.6	0.01	0.49	0.0002	NA	NA
Zhang 2015	HOTAIR	Chinese	36	median	up-regulation	0.821	0.013	0.451	0.002	0.527	0.02	0.829	NA
Zhang 2015	MALAT1	Chinese	30	NA	up-regulation	0.653	0.04	NA	0.03	NA	NA	NA	NA
Zhang 2015	MEG3	Chinese	108	NA	down-regulation	0.15	<0.01	0.16	<0.01	0.12	<0.01	NA	0.22
Cao 2016	SPRY4-IT1	Chinese	100	fold-change	up-regulation	0.068	<0.001	0.954	<0.001	0.046	<0.001	NA	NA
Kim 2016	HOXA11-AS	Korean	91	fold-change	up-regulation	0.734	NA	0.098	0.23	NA	0.142	NA	0.052
KOBAYASHI 2016	XIST	Japanese	49	median	down-regulation	0.12	0.87	NA	0.81	NA	0.11	NA	NA
Lee 2016	HOTAIR	Korean	153	fold-change	up-regulation	0.835	0.03	0.711	0.413	NA	0.043	0.732	0.037
Sun 2016	HOTAIR	Chinese	59	NA	up-regulation	0.6321	0.5132	0.0063	0.0154	NA	0.0214	NA	0.038
Wang 2016	HULC	Chinese	244	median	up-regulation	0.883	0.256	0.72	0.001	NA	NA	NA	NA
Yang 2016	PVT1	Chinese	88	NA	up-regulation	NA	<0.001	NA	<0.001	NA	<0.001	NA	NA
Zhang 2016	ANRIL	Chinese	51	median	up-regulation	0.449	0.219	0.696	0.009	0.168	0.031	NA	NA
Zhang 2016	PVT1	Chinese	90	median	up-regulation	NA	<0.01	NA	<0.01	NA	NA	NA	NA
Hu 2017	TUG1	Chinese	40	median	up-regulation	0.533	<0.001	1	0.009	0.007	0.015	0.083	NA

Abbreviations: LncRNA, long non-coding RNA; FIGO, International Federation of Gynecology and Obstetrics; NA, not available

**Figure 2:**
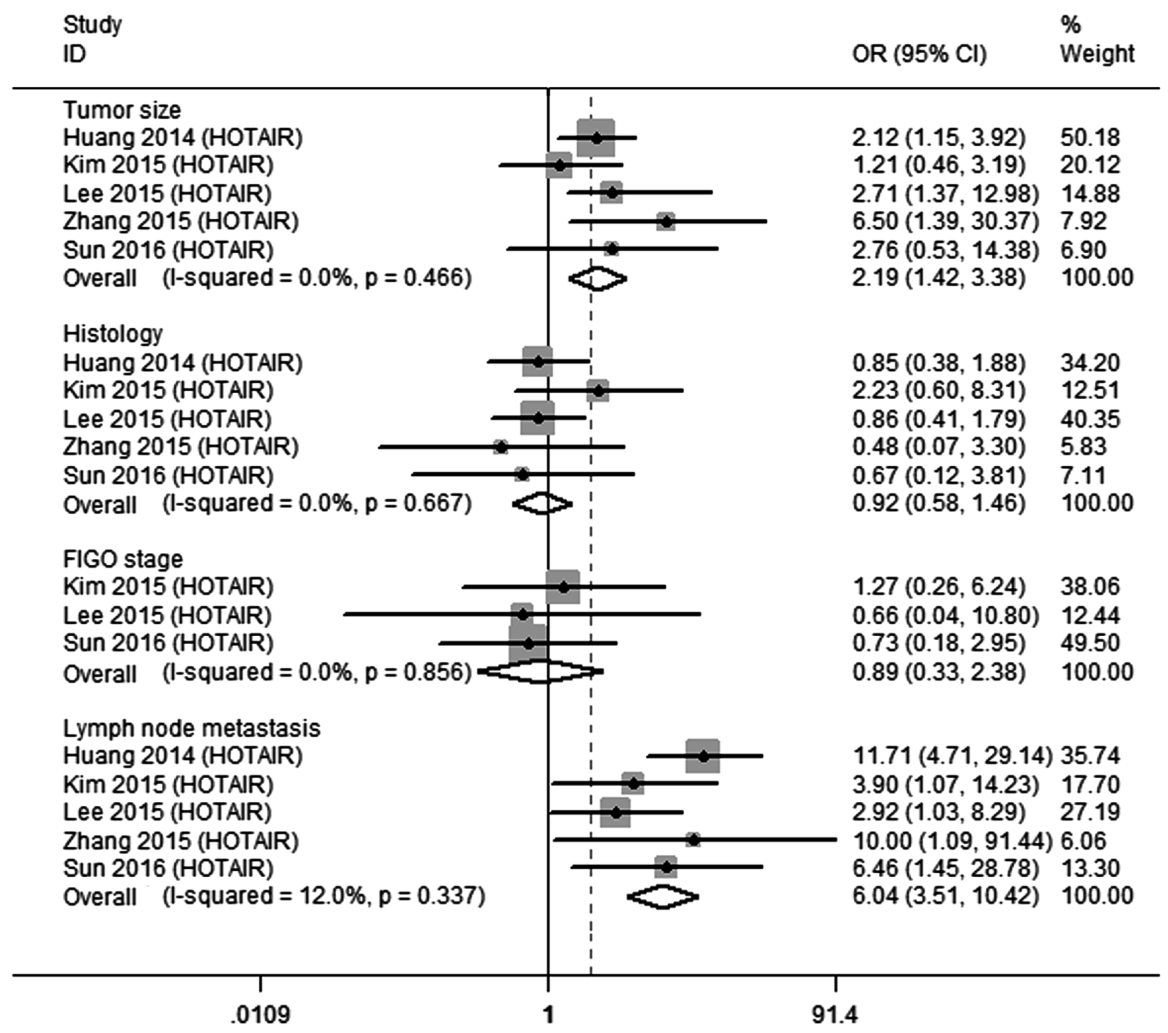
Qualitative meta-analysis of studies estimating ORs of up-regulated HOTAIR expression and the clinicopathology of CC patients Abbreviations: OR, odds ratio; CI, confidence interval.

It is worth mentioning that there were three lncRNAs (HOTAIR, MALAT1 and PVT1) investigated by at least 2 studies. MALAT1 and PVT1 were excluded because the information was incomplete. We then performed a meta-analysis to determine the possible relationship between HOTAIR overexpression and clinicopathological features. The data extracted from 5 studies (*n* = 577) [13-17] were divided into 4 groups according to different clinicopathological features. No significant heterogeneity was found (tumor size, *I*^2^ = 0.0%, *P* = 0.466; histology, *I*^2^ = 0.0%, *P* = 0.667; FIGO stage, *I*^2^ = 0.0%, *P* = 0.858; lymph node metastasis, *I*^2^ = 12.0%, *P* = 0.337) (Figure 2), and the fixed effect model was therefore utilized. The results revealed that high expression of HOTAIR was related to larger tumor size ( > 4 cm *vs* ≤ 4 cm: OR = 2.19, 95%CI 1.42-3.38, *P* = 0.000). In addition, patients with lymph node metastasis exhibited higher expression of HOTAIR than those without metastasis, with a pooled OR of 6.04 (95%CI 3.51-10.42, *P* = 0.000). Furthermore, there was no clear connection between up-regulation of HOTAIR and CC histology (adenocarcinoma [AD]/adenosquamous cell carcinom [ASC] *vs* squamous cell carcinoma [SCC]: OR = 0.92, 95%CI 0.58-1.46, *P* = 0.714) or FIGO stage (III/IV *vs* I/II: OR = 0.89, 95%CI 0.33-2.38, *P* = 0.813).

### Prognosis

**Figure 3:**
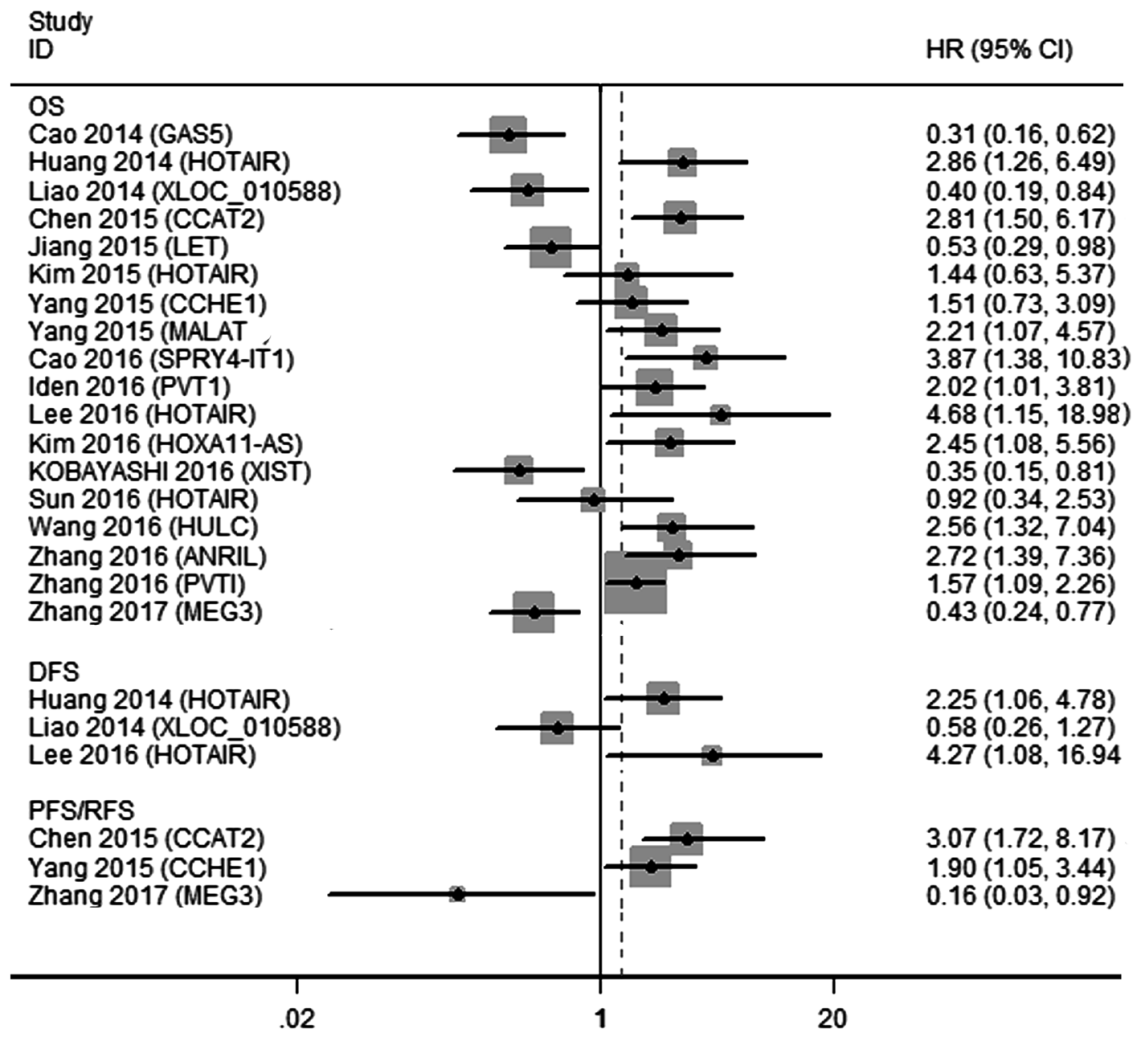
A display of HRs of lncRNAs in CC patients Abbreviations: OS, overall survival; DFS, disease-free survival; PFS, progression-free survival; RFS, recurrence-free survival; HR, hazard ratio; CI, confidence interval.

Eighteen studies containing 2291 patients were available to investigate the relationship between lncRNA expression and OS. Three studies reported DFS and PFS/RFS data. The characteristics of these eligible studies are presented in Table 2. Increased expression of HOTAIR [13-15, 17], CCAT2 [18], CCHE1 [19], MALAT1 [20], SPRY4-IT1 [22], HOXA11-AS [23], HULC [24], PVT1 [11, 25], and ANRIL [26] were associated with a poor prognosis, together with decreased expression of GAS5 [28], XLOC_010588 [12], LET [29] and MEG3 [32] (Figure 3).

**Table 2:** Summary of lncRNAs used as prognostic biomarkers of CC

Studies	LncRNAs	Population	Tumor stage	Case number		Detected sample	Detection methord	Cut-off	Outcomes	HR availability	Follow-up month
				High level	Low level						
Cao 2014	GAS5	Chinese	Ⅰb-Ⅲa	58	44	FT	qRT-PCR	median	OS	directly	44(0-60)
Huang 2014	HOTAIR	Chinese	Ⅰb-Ⅱb	109	109	FT	qRT-PCR	median	OS,DFS	directly	42(2-55)
Liao 2014	XLOC_010588	Chinese	Ⅰb-Ⅱb	109	109	FT	qRT-PCR	median	OS,DFS	directly	42(2-55)
Chen 2015	CCAT2	Chinese	Ⅰb-Ⅲa	62	61	FT	qRT-PCR	median	OS,PFS	directly	48(6-60)
Jiang 2015	LET	Chinese	Ⅰb-Ⅲa	44	50	FT	qRT-PCR	mean	OS	directly	46
Kim 2015	HOTAIR	Korean	Ⅰa-Ⅳb	89	22	FT	qRT-PCR	fold-change	OS	indirectly	∼64
Yang 2015	CCHE1	Chinese	Ⅰb-Ⅱb	91	91	FT	qRT-PCR	median	OS,RFS	indirectly	∼40
Yang 2015	MALAT1	Chinese	Ⅰb-Ⅲa	52	52	FT	qRT-PCR	median	OS,RFS	directly	30(8-60)
Cao 2016	SPRY4-IT1	Chinese	Ⅰb-Ⅱb	46	54	FT	qRT-PCR	fold-change	OS	directly	53(3-60)
Iden 2016	PVT1	American	NA	63	58	FT	qRT-PCR	median	OS	indirectly	2-120
Lee 2016	HOTAIR	Korean	Ⅰ-Ⅲ	92	61	FS	qRT-PCR	fold-change	OS,DFS	directly	55(1-99)
Kim 2016	HOXA11-AS	Korean	Ⅰ-Ⅲ	92	30	FT	qRT-PCR	fold-change	OS	directly	46.5
KOBAYASHI 2016	XIST	Japanese	Ⅰ-Ⅳ	24	25	FT	qRT-PCR	median	OS	indirectly	44.1(5.2-142.1)
Wang 2016	HULC	Chinese	Ⅰb-Ⅲa	120	124	FT	qRT-PCR	median	OS	directly	0-60
Zhang 2016	ANRIL	Chinese	Ⅰb-Ⅲa	27	26	FT	qRT-PCR	median	OS	directly	0-60
Zhang 2016	PVT1	Chinese	Ⅰ-Ⅱ	45	45	FT	qRT-PCR	median	OS	indirectly	0-58
Zhang 2017	MEG3	Chinese	Ⅰ-Ⅱ	36	36	FT	qRT-PCR	median	OS,RFS	directly	0-36

Abbreviations: LncRNA, long non-coding RNA; FT, frozen tissue; qRT-PCR, quantities reverse transcription polymerase chain reaction; OS, overall survival; DFS, disease free survival; HR, hazard ratio

**Figure 4:**
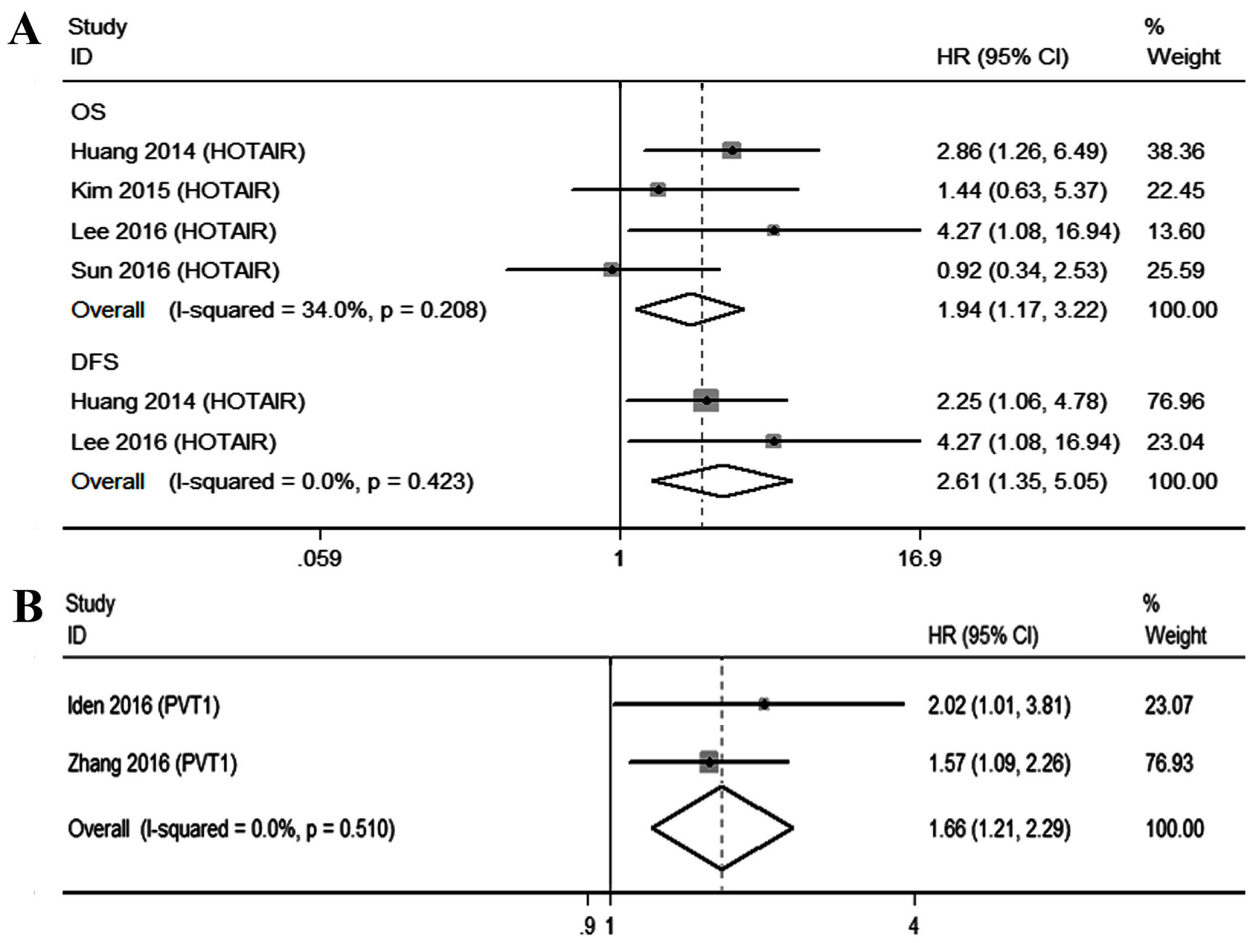
Qualitative meta-analysis of studies estimating the relationship between lncRNA expression and the prognosis of patients with CC **A.** HOTAIR **B.** PVT1. Abbreviations: OS, overall survival; DFS, disease-free survival; HR, hazard ratio; CI, confidence interval.

Two lncRNAs (HOTAIR and PVT1) were investigated in at least 2 studies, and we performed meta-analyses of the survival data. For HOTAIR, four studies (*n* = 541) described the relationship between expression and outcome in CC patients, including 4 on OS (*n* = 541) and 2 on DFS (*n* = 371). We then incorporated these studies with OS and DFS separately. Fixed effects models were applied because the heterogeneity was not significant (OS, *I*^2^ = 34.0%, *P* = 0.208; DFS, *I*^2^ = 0.0%, *P* = 0.424). The results revealed that high expression of HOTAIR was a predictive factor of shorter OS (HR, 1.94, 95%CI 1.17-3.22, *P* = 0.011), as well as DFS (HR, 2.61, 95%CI 1.35-5.05, *P* = 0.004) (Figure 4A). It is worth noting that before reintegration was performed, up-regulation of HOTAIR was independent of OS according to the clinical data given by the two studies [14, 17]. However, the results of our pooled analysis indicated HOTAIR as a prognostic factor.

As for PVT1, two studies (*n* = 211) assessed that raised PVT1 levels yielded a worse OS in CC patients. Because of the low heterogeneity (*I*^2^ = 22.2%, *P* = 0.510), the fixed effects model was used. The subsequent combined adjusted HR for PVT1 was 1.66 (95%CI 1.21-2.29, *P* = 0.002) (Figure 4B).

### Diagnosis

**Figure 5:**
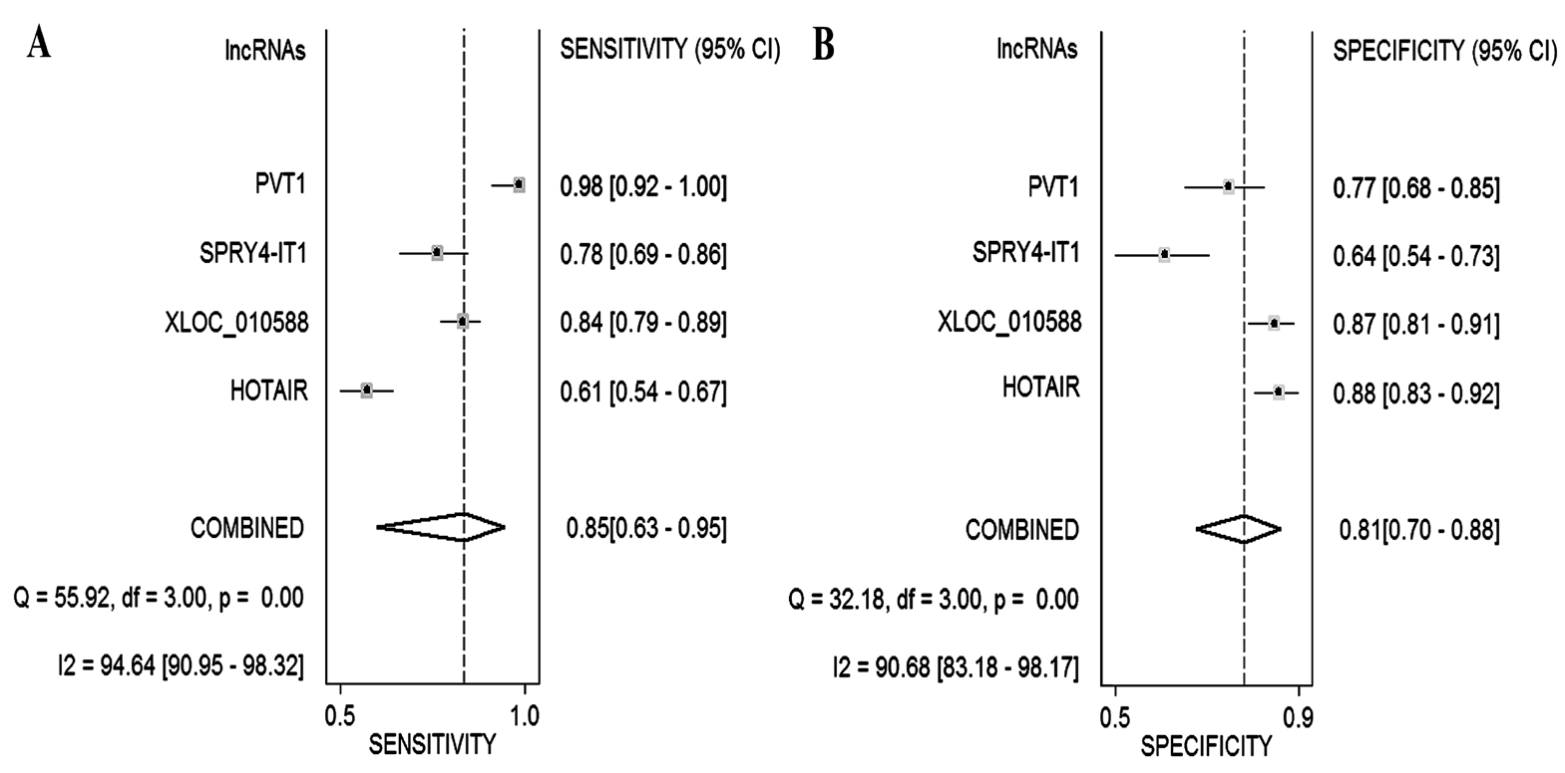
Forest plot of sensitivity and specificity of lncRNAs for the diagnosis of CC **A.** sensitivity **B.** specificity.

**Figure 6:**
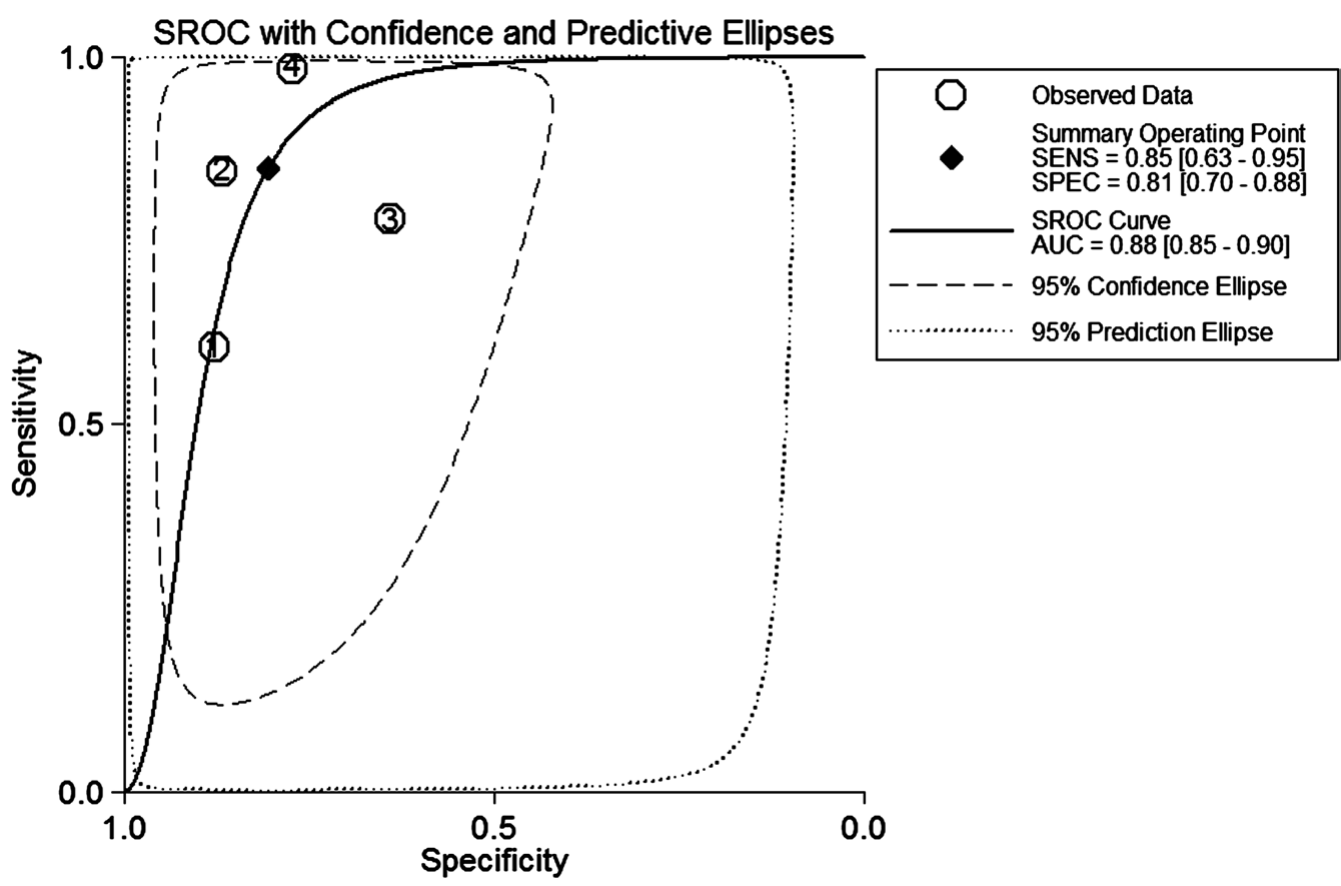
The summary receiver operator characteristic (SROC) curve based on all lncRNAs Abbreviations: SECS, sensitivity; SPEC, specificity; AUC, area under the curve.

Only 4 studies, discussing XLOC_010588 [12], HOTAIR [13], SPRY4-IT1 [22] and PVT1 [25], provided complete diagnosis-related data, among which, 3 were based on cervical tissues as specimens and 1 on serum (Table 3). Forest plots of the sensitivity and specificity of lncRNA for diagnosing CC are displayed in Figure 5. A significant heterogeneity was observed (*I*^2^ = 94.64% and *I*^2^ = 90.68%), and thus, a more conservative random effect model was used. The summary estimates are as follows: sensitivity (SEN), 0.85 (95%CI 0.63-0.95); specificity (SPE), 0.81 (95%CI 0.70-0.88); positive likelihood ratio (PLR), 4.37 (95%CI 2.83-6.74); negative likelihood ratio (NLR), 0.19 (95%CI 0.07-0.50); and overall diagnostic odds ratio (DOR), 23.18 (95%CI, 7.19-74.70). In addition, we generated a summary receiver operator characteristic (SROC) curve (Figure 6) and calculated the area under the curve (AUC) (0.88, 95%CI 0.85-0.90). These results suggested that lncRNAs achieved a relatively high diagnostic accuracy. Although large heterogeneity in this analysis was noted, we did not conduct meta-regression or subgroup analysis due to the small numbers and small sample sizes of the included studies. Therefore, further studies should be performed to verify this conclusion.

**Table 3:** Summary of lncRNAs used as diagnostic biomarkers of CC

Studies	LncRNAs	Population	Expression	Detected sample	SE(%)	SP(%)	AUC	Sample size		QUADAS-2 scores
								cancer	control	
Huang 2014	HOTAIR	Chinese	up-regulation	FT	60.60%	87.20%	0.803	218	218	5
Liao 2014	XLOC_010588	Chinese	down-regulation	FT	84.40%	86.70%	0.918	100	100	5
Cao 2016	SPRY4-IT1	Chinese	up-regulation	FT	78.30%	63.60%	0.741	218	218	5
Yang 2016	PVT1	Chinese	up-regulation	Serum	71.60%	98.80%	0.932	88	86	5

Abbreviations: LncRNA, long non-coding RNA; FT, frozen tissue; SE, sensitivity; SP, specificity; AUC, area under the curve; QUADAS, quality assessment of diagnostic accuracy studies

### Publication bias

**Figure 7:**
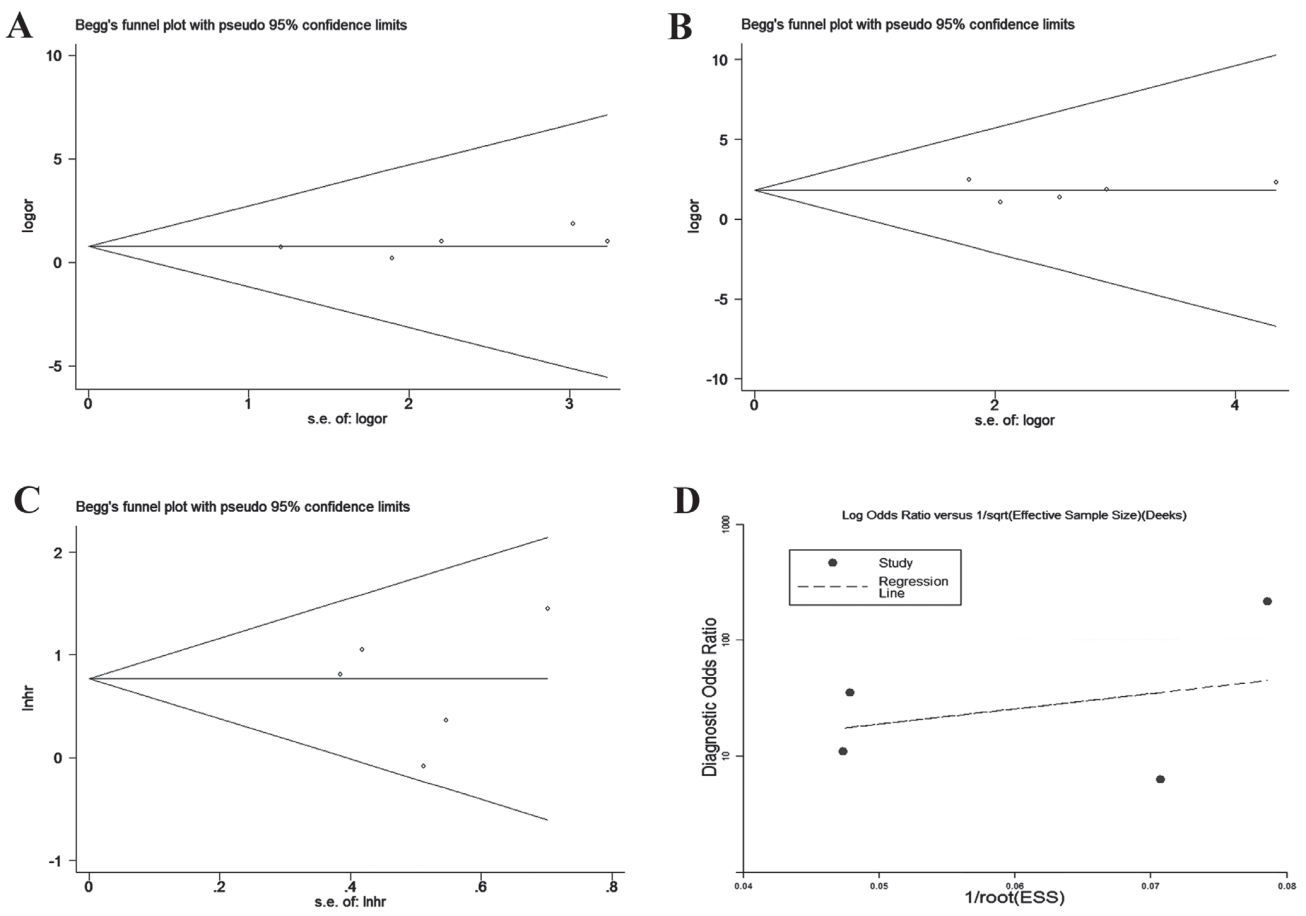
Begg’s and Deeks’ funnel plot for studies involved in the meta-analysis of HOTAIR expression and the clinical values of patients with CC **A.** Tumor size **B.** Lymph node metastasis **C.** Prognosis **D.** Diagnosis.

In the present meta-analysis, we utilized Begg’s and Egger’s tests [33], as well as funnel plots, to evaluate the publication bias of the incorporated studies. As presented in Figure 7, our analyses suggested no evident asymmetry publication bias with regard to the studies on HOTAIR using Egger’s test (*P* = 0.406 for tumor size, *P* = 0.927 for lymph node metastasis, and *P* = 0.922 for OS). For PVT1, no conclusive graph could be generated due to the small size of the associated studies, and we therefore did not evaluate publication bias. For diagnostic studies, a Deeks’ funnel plot asymmetry test [34] was conducted, and there was no clear evidence of publication bias (*P* = 0.51) in this meta-analysis.

## DISCUSSION

Within a relatively short period of time, accumulating studies have indicated that lncRNAs are frequently abnormally expressed in CC. These lncRNAs are likely to serve as diagnostic and prognostic biomarkers and may be potential targets for individualized therapy, but the relatively small sample sizes and noisiness of microarray data have produced inconsistent biological conclusions. The systematic review and meta-analysis presented here is the first comprehensive description of independent profiling experiments investigating the effect of lncRNA expression on the clinical values of CC.

In this study, we examined the correlation between lncRNAs and the main clinicopathological characteristics of CC. The results revealed that patients with dysregulated lncRNA expression were more likely to have a high histological grade and FIGO stage, lymph node metastasis deep cervical invasion and large tumor size. Among the included publications, HOTAIR was the most widely investigated lncRNA, as it was reported in 5 studies. The pooled data illustrated that HOTAIR expression was remarkably correlated with tumor size and lymph node metastasis in CC patients. Unsurprisingly, a recent meta-analysis of lncRNA in all human cancers [35] has some overlap with our pooled analysis, specifically, increased HOTAIR expression is often found in cancer patients with lymph node metastasis compared to those without metastasis (OR = 2.81, 95%CI 1.38-5.70, *P* = 0.004, random-effects model). From this point of view, these analyses provide a promising way for determining whether HOTAIR functions as a biomarker for lymph node metastasis in CC patients.

For the prognostic values, most lncRNAs were identified by a single study, while only two (HOTAIR and PVT1) were reported by at least two studies. Our subsequent pooled data analyses discovered that high expression of HOTAIR is a strong predictor of short OS and DFS of CC patients. Consistent with the present meta-analysis, Miao et al. [36] pooled 63 studies with various solid carcinomas and found that a high level of HOTAIR predicted worse OS with a combined HR of 2.21 (95%CI 1.77-2.74, *P* < 0.00001). In the subgroup analysis, higher levels of HOTAIR also indicated shorter OS in Asian populations (HR = 2.06, 95%CI 1.80-2.37, *P* < 0.00001). In general, high HOTAIR expression represents a significant risk factor for survival outcomes in the development of tumors. In addition, an increase in cellular expression of PVT1 was significantly associated with a decrease in overall survival. It follows that up-regulation of HOTAIR and PVT1 could be considered prognostic markers for CC.

Regarding the diagnostic values, it was unlikely that an accurate assessment of the diagnostic performance of lncRNAs could be obtained due to the small sample sizes in the included studies. Therefore, the aim of our study was to summarize the results of individual studies and investigate the diagnostic value of lncRNAs for CC detection. After analyzing and pooling all of the included data, we found that the overall sensitivity and specificity of lncRNAs were 0.85 and 0.81 with an AUC value of 0.88. Nevertheless, we were unable to perform advanced analysis because of the limited and insufficient research regarding other lncRNAs.

For one of the most extensively studied lncRNAs, HOTAIR, a high expression level is observed in many malignancies [37-39]. Consistent with our results, a number of studies have demonstrated that HOTAIR expression is related to clinical parameters and the prognosis of cancer patients. The biological role of HOTAIR in tumor cells may mediate the poor CC outcome. HOTAIR was introduced by Rinn et al. [40] as a spliced and polyadenylated RNA with 2,158 nucleotides and 6 exons. It functions as a molecular scaffold and interacts with polycomb repressive complex 2 (PRC2) and lysinespecific demethylase1 (LSD1) complex to regulate gene expression. Approximately 854 genes with HOTAIR-induced PRC2 occupancy are implicated in inhibiting breast cancer progression, including classic favorable prognostic factors [41]. In addition, Padua et al. [42] have discovered a close connection between HOTAIR and epithelial-mesenchymal transition (EMT) and its role in inducing and maintaining cancer stem cells (CSCs). Furthermore, HOTAIR can function as a competitive endogenous RNA (ceRNA) in cancer cells, recruiting microRNAs to target various genes [43, 44].

Still, there are several limitations in identifying a correlation between aberrant expression of lncRNA and clinical values in the present meta-analysis. First, the arbitrary cut-off value for low or high levels of lncRNA in the patient samples differed between studies. Though qRT-PCR was used in all of the studies to quantify lncRNAs, the results may still be heterogeneous due to the utilization of different qRT-PCR primer sets across studies of the same lncRNA. Second, obstacles in achieving a sufficient follow-up period and homogenous endpoints limited the accuracy of the results. Third, the method of HR extrapolation from the Kaplan-Meier graph may also generate heterogeneity despite analysis by two independent reviewers to minimize this variation. Five of the records included in the systematic review did not report the HR directly. Therefore, the extrapolated HRs might be less reliable compared with reported statistics. Fourth, in this systematic review, most of the studies addressed different lncRNAs. Only 4 lncRNAs (HOTAIR, MALAT1, PVT1 and MEG3) were identified by at least two studies. Furthermore, the majority of patients are Asian, only one study with American patients. Therefore, most of the meta-analyses in our study contain insufficient records. Finally, a distinct heterogeneity was observed in the analysis of diagnostic value. Due to the small number and small sample sizes of the included studies, we did not conduct a meta-regression or meta-subgroup analyses.

To sum up, the strong clinical value of lncRNA expression in CC was confirmed in the present results, especially HOTAIR, a promising potential biomarker for lymph node metastasis and survival rate in cancer patients. Furthermore, lncRNA also exhibited appropriate accuracy for CC diagnosis. Further, more comprehensive and large-scale studies are required to achieve a more persuasive conclusion.

## MATERIALS AND METHODS

### Literature search strategies

The aim of our systematic review and meta-analysis was to identify all the primary research articles that assessed the utility of candidate lncRNAs as biomarkers for clinical values in CC. A comprehensive search was performed in PubMed (Supplementary Table 3), Embase and Web of Science databases prior to February 10, 2017.

### Inclusion and exclusion criteria

All of the included studies had to meet the following inclusion criteria: (1) the association between lncRNAs and cervical cancer was discussed with regard to clinicopathological features, prognostic or diagnostic values; (2) all the cancer patients were diagnosed based on a histopathological or cytological examination, considered the gold standard for diagnosis, and lncRNA expression in tumors or blood samples was estimated in the study; (3) studies provided sufficient data for extraction or calculation of the individual OR, HR and 95%CI. Studies were excluded based on the following criteria: (1) duplicate publications; (2) reviews, letters, laboratory studies and meeting abstracts; (3) fewer than 30 sample cases; (4) studies without complete data.

### Data extraction and quality assessment

Eligible publications were reviewed independently by two investigators. The following data were extracted: basic information of included records, characteristics of the patients, and essential data for systematic review and meta-analysis. When the HR and 95%CI for survival analysis were unavailable, we calculated the HRs and their 95%CIs using Kaplan-Meier curves and observed data provided by the authors based on the methods illustrated by Tierney et al. [45]. The methodological quality of prognostic studies was evaluated using the Newcastle-Ottawa-Scale (NOS) tool [46]. The NOS score has a maximum of nine and those studies ≥7 were considered to be of high quality. Moreover, QUADAS-2 [47] was adopted to assess the quality of all the included diagnostic studies. The QUADAS-2 tool comprises four key domains: patient selection, index test, reference standard, flow and timing, and judge bias and applicability. This is an evidence-based tool for quality assessment intended for use with diagnostic accuracy studies, with a maximum score of seven.

### Statistical analysis

Meta-analyses were performed using Stata version 12.0 (StataCorp, College Station, TX, USA). A different effect size (ES) was selected for each meta-analysis. (1) Pooled HRs and ORs with 95%CIs were used to evaluate the association between lncRNA expression and CC prognosis and clinicopathological features. The HRs and 95%CIs were directly extracted from original articles or estimated from the existing data using methods previously reported by Tierney et al. [45]. An observed HR > 1 implied a worse survival for patients with up-regulated lncRNA expression. Conversely, an HR < 1 implied a worse survival for patients with decreased lncRNA expression [48]. The point estimate of the HR or OR was considered statistically significant at a level of *P* < 0.05 if the 95%CI did not cover the value “1”. (2) Sensitivity, specificity, PLR, NLR, DOR, SROC curve, and AUC were used for the diagnostic meta-analysis. A heterogeneity test was conducted using Cochran’s Q test and Higgins I-squared statistic. *I*^2^ values > 50% indicated heterogeneity among studies [49]. When heterogeneity was observed (*I*^2^ > 50%), a random-effect model was used; otherwise, a fixed-effect model was used. Furthermore, publication bias was assessed by visual inspection and statistically evaluated by Begg’s and Deeks’ funnel plot, using Egger’s test. Asymmetric funnel plots or *P* < 0.05 in Egger’s test suggest the existence of publication bias in the incorporated studies.

## SUPPLEMENTARY MATERIALS TABLES


