# Correction: Prognostic and diagnostic significance of lncRNAs expression in cervical cancer: a systematic review and meta-analysis

**DOI:** 10.18632/oncotarget.22570

**Published:** 2017-11-21

**Authors:** Shuqi Chi, Lina Shen, Teng Hua, Shuangge Liu, Guobing Zhuang, Xiaoxiao Wang, Xing Zhou, Guozhen Wang, Hongbo Wang

**Affiliations:** ^1^ Department of Obstetrics and Gynecology, Union Hospital, Tongji Medical College, Huazhong University of Science and Technology, Wuhan 430022, China

**This article has been corrected:** the affiliation was updated and is as follows:

^**1**^ Department of Obstetrics and Gynecology, Union Hospital, Tongji Medical College, Huazhong University of Science and Technology, Wuhan 430022, China

Original article: Oncotarget. 2017; 8:79061-79072. https://doi.org/10.18632/oncotarget.18323

