# Supplementary material for: Prognostic and diagnostic significance of lncRNAs expression in cervical cancer: a systematic review and meta-analysis
Source: Oncotarget. 2017 May 31;8(45):79061–72. doi: 10.18632/oncotarget.18323 (PMC5668020; doi:10.18632/oncotarget.18323)
Supplement: Supplementary file 1 [file oncotarget-08-79061-s001.pdf]

Prognostic and diagnostic significance of lncRNAs expression in cervical cancer: a systematic review and meta-analysis

SUPPLEMENTARY MATERIALS

Supplementary Table 1: Newcastle-Ottawa Scale to assess the quality of the included studies

study	Selection				Comparability	Outcome			
	Representative of cases	Selection of controls	Ascertainment of exposure	Outcomes present at start of study	Comparability the design or analysis	Assessment of outcome	Adequate follow-up time (≥ 1 year)	Adequacy of follow up (≥ 80%)	Total scores
Cao 2014 (GAS5)	1	1	1	1	2	1	1	1	9
Huang 2014 (HOTAIR)	1	1	1	1	2	1	1	0	8
Liao 2014 (XLOC_010588)	1	1	1	1	2	0	1	0	7
Chen 2015 (CCAT2)	1	1	1	1	2	0	1	1	8
Jiang 2015 (LET)	1	1	1	1	2	1	1	0	8
Kim 2015 (HOTAIR)	1	1	1	1	2	0	1	1	8
Yang 2015 (CCHE1)	1	1	1	1	2	1	1	1	9
Yang 2015 (MALAT1)	1	1	1	1	2	0	1	1	8
Cao 2016 (SPRY4-IT1)	1	1	1	1	2	1	1	1	9
Iden 2016 (PVT1)	1	1	1	1	2	1	1	1	9
Lee 2016 (HOTAIR)	1	1	1	1	2	0	1	0	7
Kim 2016 (HOXA11-AS)	1	1	1	1	2	0	1	0	7
KOBAYASHI 2016 (XIST)	1	1	1	1	2	1	1	1	9
Sun 2016 (HOTAIR)	1	1	1	1	2	1	1	0	8
Wang 2016 (HULC)	1	1	1	1	2	0	1	1	8
Zhang 2016 (ANRIL)	1	1	1	1	2	0	0	0	6
Zhang 2016 (PVTI)	1	1	1	1	2	1	1	0	8
Zhang 2017 (MEG3)	1	1	1	1	2	1	1	1	9

Supplementary Table 2: The Quality Assessment of Diagnostic Accuracy Studies-2 to assess the quality of the included studies

Study	Risk of Bias				Applycability Concerns			Total scores
	Patient Selection	Index Test	Reference Standard	Flow and Timing	Patient Selection	Index Test	Reference Standard	
Huang 2014 HOTAIR	1	0	1	1	0	1	1	5
Liao 2014 XLOC_010588	1	0	1	1	0	1	1	5
Cao 2016 SPRY4-IT1	1	0	1	1	0	1	1	5
Yang 2016 PVT1	1	0	1	1	0	1	1	5

Supplementary Table 3: Literature search strategy to identify papers for the meta-analysis that describe an association between LncRNA expression and Cervical Cancer

Keywords	Search algorithm	Results
**LncRNA**	"RNA, Long Noncoding"[Mesh] OR lncRNA[Title/Abstract] OR Long ncRNA[Title/Abstract] OR ncRNA, Long[Title/Abstract] OR RNA, Long Non-Translated[Title/Abstract] OR Long Non-Translated RNA[Title/Abstract] OR Non-Translated RNA, Long[Title/Abstract] OR RNA, Long Non Translated[Title/Abstract] OR Long Non-Coding RNA[Title/Abstract] OR Long Non Coding RNA[Title/Abstract] OR Non-Coding RNA, Long[Title/Abstract] OR RNA, Long Non-Coding[Title/Abstract] OR Long Non-Protein-Coding RNA[Title/Abstract] OR Long Non Protein Coding RNA[Title/Abstract] OR Non-Protein-Coding RNA, Long[Title/Abstract] OR RNA, Long Non-Protein-Coding[Title/Abstract] OR Long Noncoding RNA[Title/Abstract] OR RNA, Long Untranslated[Title/Abstract] OR Long Untranslated RNA[Title/Abstract] OR Untranslated RNA, Long[Title/Abstract] OR Long ncRNAs[Title/Abstract] OR ncRNAs, Long[Title/Abstract] OR Long Intergenic Non-Protein Coding RNA[Title/Abstract] OR Long Intergenic Non Protein Coding RNA[Title/Abstract] OR LincRNAs[Title/Abstract] OR LINC RNA[Title/Abstract]	16233
**Cervical Cancer**	"Uterine Cervical Neoplasms"[Mesh] AND Cervical Neoplasm, Uterine[Title/Abstract] OR Cervical Neoplasms, Uterine[Title/Abstract] OR Neoplasm, Uterine Cervical[Title/Abstract] OR Neoplasms, Uterine Cervical[Title/Abstract] OR Uterine Cervical Neoplasm[Title/Abstract] OR Neoplasms, Cervical[Title/Abstract] OR Cervical Neoplasms[Title/Abstract] OR Cervical Neoplasm[Title/Abstract] OR Neoplasm, Cervical[Title/Abstract] OR Neoplasms, Cervix[Title/Abstract] OR Cervix Neoplasms[Title/Abstract] OR Cervix Neoplasm[Title/Abstract] OR Neoplasm, Cervix[Title/Abstract] OR Cancer of the Uterine Cervix[Title/Abstract] OR Cancer of the Cervix[Title/Abstract] OR Cervical Cancer[Title/Abstract] OR Uterine Cervical Cancer[Title/Abstract] OR Cancer, Uterine Cervical[Title/Abstract] OR Cancers, Uterine Cervical[Title/Abstract] OR Cervical Cancer, Uterine[Title/Abstract] OR Cervical Cancers, Uterine[Title/Abstract] OR Uterine Cervical Cancers[Title/Abstract] OR Cancer of Cervix[Title/Abstract] OR Cervix Cancer[Title/Abstract] OR Cancer, Cervix[Title/Abstract] OR Cancers, Cervix[Title/Abstract]	96896
**LncRNA AND Cervical Cancer**		93
